# Supplementary material for: Are carbon emissions trading and green financial instruments synergistic? -Comprehensive quantitative research based on content analysis
Source: PLoS One. 2024 Mar 7;19(3):e0298601. doi: 10.1371/journal.pone.0298601 (PMC10919601; doi:10.1371/journal.pone.0298601)
Supplement: S1 Data — (ZIP) [file pone.0298601.s001.zip › Data/地级市Z_F工作报告.zip!/╔╜╢1⁄2/╟α╡║╩╨/└┤╫╘╣1⁄2╓┌║┼í╛┬φ┐╦╩2╛▌═°í┐_2009.docx]

各位代表：

　　现在，我代表青岛市人民政府，向大会作工作报告,请予审议，并请各位政协委员和其他列席人员提出意见。

　　一、2008年工作回顾

　　2008年，是青岛发展进程中很不寻常、很不平凡的一年。一年来，我们经历了历史罕见、难以预料的重大挑战和考验。在中共青岛市委的坚强领导下，我们紧紧依靠全市人民，全面贯彻党的十七大和胡锦涛总书记视察青岛重要讲话精神，深入落实科学发展观，认真执行国家和省的决策部署，适时调整工作重点，沉着应对各种困难，成功举办了一届有特色、高水平的奥帆赛、残奥帆赛，经济保持平稳较快发展，社会保持和谐稳定，完成了市十四届人大一次会议确定的目标任务。

　　初步统计，全市生产总值迈上4000亿元台阶，达到4436.2亿元，比上年增长13.2%；地方财政一般预算收入342.4亿元，增长17%；城市居民人均可支配收入20464元，农民人均纯收入8509元，分别增长14.6%和13.8%；居民消费价格指数上升4.7%；城镇登记失业率3.01%；人口自然增长率2.62％。。万元生产总值能耗和二氧化硫、化学需氧量排放量持续下降。

　　（一）积极应对复杂多变的宏观形势，经济保持平稳较快发展

　　一年来，我们牢牢把握发展第一要务，坚持一手抓经济社会发展，一手抓奥帆赛、残奥帆赛筹办，审时度势，周密部署，努力增强经济工作的预见性、针对性和实效性，保持了经济发展的稳定性、协调性和持续性。

　　年初，按照国家宏观调控的要求，推进重点项目建设，稳定市场物价，实现了良好开局。年中，针对经济增速放缓的突出问题，及时出台应对出口下滑、解决中小企业经营困难、促进服务业发展等措施，保持了经济平稳运行。进入四季度，根据国家扩内需保增长的部署，迅速制定12条实施意见，集中安排了到2010年的500个重点项目，总投资超过5000亿元。城市地铁建设规划上报国家审批，环胶州湾高速公路市区段拓宽改造等一批工程开工建设。组织银企贷款授信和工贸供需对接，加快了出口退税进度。出台鼓励居民购房政策，稳定了房地产市场。取消、停征或降低一批行政事业收费和经营性收费，减轻了企业负担。通过采取一系列有力措施，有效遏制了经济增速明显回落的态势，为完成全年目标任务创造了条件、奠定了基础。

　　结构调整和自主创新取得新成效。一批事关青岛发展的重大项目加快推进。年初确定的71个重点项目全部开工，竣工投产或主体竣工32个。1000万吨大炼油、通用五菱30万辆微型车、海西湾50万吨级造船坞等项目建成投产。前湾港四期集装箱码头、青岛港液体化工码头、香港招商局集装箱码头建成启用。规模以上固定资产投资超过2000亿元，增长23.5%；其中，服务业投资突破1000亿元，增长31.4%。规模以上工业增加值和工业总产值分别超过2000亿元、8000亿元。港口货物吞吐量和集装箱吞吐量突破3亿吨、1000万标箱。空港旅客吞吐量和货邮吞吐量超过800万人次、13万吨。港中旅海泉湾等16个旅游项目开工建设，海尔洲际酒店、海信广场等一批服务设施竣工开业。引进金融机构8家，新增本外币存贷款861亿元和688亿元，保费收入突破百亿元。社会消费品零售总额增长22.2%。服务业增加值占生产总值比重达到44.1%，提高1.1个百分点。高速动车组、液晶背光模组等技术攻关实现突破，高新技术产业产值增长20.1%。建成中科院生物能源与过程研究所。发明专利申请量和授权量分别增长62.9%、23.5%。成功举办了首届中国国际循环经济成果交易博览会。

　　“环湾保护、拥湾发展”战略全面启动。完成新一轮城市总体规划修编。高新区胶州湾北部园区和环湾重要节点建设全线展开，老城区企业搬迁改造有序推进，县域6个工业功能区和董家口港区加快规划建设。青岛火车站、长途汽车站扩建改造和快速路三期跨线桥等工程相继完工。楼山河、李村河等污水处理项目建成使用，污水处理能力提高50%。市区空气质量一级天数增加40%。荣获“中华宝钢环境奖”。实施了迎奥运亮化绿化美化工程，数字化城市管理指挥系统基本建成。

　　新农村建设扎实推进。市级财政投入“三农”资金21.9亿元，增长52%。农牧渔业全面丰收，粮食总产达到333.7万吨，创十年来最好水平。完成100座小型病险水库除险加固。46%的行政村实现“五化”目标。农村学校现代远程教育工程全部建成。免费为农村义务教育阶段学生提供教科书。镇级卫生院标准化建设全面完成，新型农村合作医疗为农民报销医药费4.1亿元，比上年增加66%。改扩建村文化活动室1200处，新建农家书屋1000个。落实“家电下乡”补贴政策，发放补贴1350万元。县域生产总值、财政收入增幅均高于全市平均水平。农村经济社会的快速发展，为增加农民收入、保障市场供应、促进城乡和谐稳定发挥了重要作用。

　　改革开放继续深化。以纪念改革开放30周年为契机，加快重点领域改革步伐。政府经营类投资体制改革实现突破，整合组建了国信、城投、华通三大投资集团。完成黄岛电厂产权重组，实现了与大唐集团的战略合作。纺织、机械等行业调整重组取得积极进展。理顺了市与区（市）的事权和财权。设立中小企业发展专项资金和融资服务中心。民营经济增加值增长15%左右。全年完成出口退税178亿元。进出口总额和出口总额分别突破500亿美元、300亿美元。实际到账外资26.4亿美元，增长10%。引进内资突破400亿元。前湾保税港区获国家批准。青岛与台湾实现海空直航。日本驻青总领事馆开馆。与美国达拉斯等5城市结为友好关系城市。

　　（二）全面战胜浒苔自然灾害，成功举办奥帆赛、残奥帆赛

　　一年来，我们把成功举办一届有特色、高水平奥帆赛、残奥帆赛作为重中之重，举全市之力，集各方之智，付出了艰苦卓绝的努力，兑现了郑重承诺，实现了奥运梦想。

　　赛前筹备周密到位。对赛事组织、安全保卫、奥运村运行、比赛海域管理等进行了综合演练。成功举办青岛国际残疾人帆船赛，残奥设施和服务得到全面检验。奥运火炬接力青岛站传递圆满出色，成为全国秩序最好、气氛最热烈的城市之一。

　　六月，青岛近海遭遇了突如其来、历史罕见的浒苔自然灾害，使即将到来的奥帆赛、残奥帆赛受到严重威胁。危急关头，胡锦涛总书记作出重要批示，并亲临青岛视察指导，给我们极大鼓舞。在省委、省政府和市委的坚强领导下，各方紧急支援，军地联合作战，科技有力保障，万众一心、众志成城，夺取了抗击浒苔自然灾害的全面胜利，确保了帆船比赛如期顺利举行。

　　赛事举办精彩成功。进入赛时，建立了强有力的运行总指挥体系，形成全民动员、全民参与的工作机制。社会各界、驻青单位、高等院校和人民解放军、武警部队、公安干警团结拼搏，勇挑重担，以科学的赛事组织、严密的安全保卫、高效的城市运行、热情的志愿服务、规范的礼宾接待、良好的媒体宣传、优美的城市环境，实现了让国际社会满意、让各国运动员满意、让人民群众满意的目标。胡锦涛总书记就青岛为北京奥运会、残奥会所做的突出贡献作出重要批示，给予充分肯定。北京奥组委和国际奥委会、残奥委会对赛事的成功给予高度评价。我市12个单位、10名同志受到党中央、国务院的表彰，22个单位、47名同志被省委、省政府授予先进集体和先进个人荣誉称号。

　　各位代表！经过7年的艰辛筹备，青岛人民向世界展现了一幅千帆竞发、激情飞扬的壮丽画卷，向祖国交上了一份合格答卷。奥帆赛、残奥帆赛的成功举办，不仅展示了富强文明和谐的城市风采，为现代帆船运动增添了青岛元素；也为百年青岛树立了新的里程碑，留下了丰厚的奥运遗产。奥运效应正在加快推动城市面貌日新月异，奥运文化正在全面提升城市文明程度，奥运精神正在成为促进青岛长期繁荣发展的强大动力！

　　（三）更加注重改善民生，社会环境保持和谐稳定

　　一年来，我们顺应广大市民的新期待，把保障和改善民生作为工作的出发点和落脚点，加大民生投入，使城乡居民生活水平进一步提高。

　　尽心竭力为群众办实事。对人民群众承诺的12件实事全部兑现。积极应对就业形势变化，出台了失业预警应急预案，规范企业裁员行为，城乡就业保持稳定。全市财政投入2.7亿元，确保了7.5万户城乡困难家庭的基本生活。投入2.2亿元，资助了14万名家庭经济困难学生。实施了物价上涨与困难群众生活补贴联动机制。低保家庭取暖补贴标准大幅提高。市财政投入5亿元，新建廉租住房3300套，整治改造筒子楼149栋、旧住宅区4片，受益居民1.6万户。改造旧城区、城中村21片，惠及居民1.7万户，配建经济适用住房5000套。到年底，为市区3.5万户低收入住房困难家庭提供保障性住房9400套，实物配租、配售住房达到27%，享受廉租住房租金补贴的家庭实现应保尽保。市区新增集中供热面积520万平方米，普及率提高到52%。中山公园、植物园和公共博物馆、图书馆、艺术馆等免费开放。

　　社会事业全面进步。出台了推动文化大发展大繁荣的政策措施，文化体制改革迈出重要步伐。文化产业实现增加值210亿元。“蔚蓝青岛”文化品牌初步形成，国际小提琴比赛、国产电影交易会成功举行，奥运文化广场演出等群众性文化活动蓬勃开展。国医堂、妇女儿童医疗保健中心开工建设。健全公共卫生体系，全市无重大疫情发生。完成54所市区中小学取暖设施改造。职业教育现代制造业实训基地投入使用，第一国际学校开工建设。高等教育协调服务机制进一步完善，产学研结合和人才培养取得新成果。市科技馆完成选址工作。市体育馆基本建成，游泳跳水馆完成主体工程。青岛籍运动员在北京奥运会上夺得4枚奖牌，实现了我市奥运金牌零的突破；在残奥会上取得1金1银的好成绩。制定实施促进残疾人事业发展的意见，尊重关爱残疾人的社会氛围更加浓厚。老龄工作不断加强，养老服务“双千计划”超额完成。认真做好人口计划生育工作，低生育水平持续稳定。第二次全国经济普查工作顺利实施。

　　文明和谐创建不断加强。深入推进人文奥运工程，营造了开放包容、文明和谐的城市氛围。被中央文明办授予“全国未成年人思想道德建设工作先进城市”称号，连续第六次获得国家无偿献血城市奖。完善专群结合的社会防控体系，广泛开展“大接访”活动，集中解决了一批群众反映的突出问题。切实做好安全生产工作，“隐患治理年”活动扎实推进。加强了食品药品质量监管。认真做好“双拥”工作，加强国防动员、人民防空和国防后备力量建设，军政、军民关系更加密切。

　　汶川特大地震发生后，我们迅速组织力量参与抢险救援，积极做好灾区伤员救治、学生就学安置和援川重建工作。全市人民心系灾区，累计捐赠款物6.1亿元。救灾款物管理使用做到公开透明。提前完成过渡性安置房建设任务，编制了两个对口援建乡镇的修建性规划，援建工作取得阶段性重要成果，得到中央、省和灾区人民的高度赞誉。

　　（四）不断加强政府自身建设，行政服务效能明显提高

　　一年来，我们围绕建设服务型政府的目标，全面履行政府职责，加快转变政府职能，积极推进服务创新，政务环境不断改善。

　　依法行政能力进一步增强。坚持重大事项向人大报告和向政协通报制度。人大代表议案、建议和政协委员提案全部办复。提请市人大常委会审议通过地方性法规3件，制定和修改政府规章5件。加强了政府部门与各民主党派、工商联的对口联系。充分发挥市长热线、网上访谈、行风在线等作用，自觉接受社会监督。修订完善了《青岛市人民政府工作规则》，信息公开和行政问责制初步建立。举行政府新闻发布73次，比上年增加43%。市行政审批服务大厅试运行，网上审批及电子监察系统建成。

　　勤政廉政建设进一步加强。政府应急处置能力明显提高，城市公共安全应急管理体系更加完善,在抗击自然灾害、处置突发事件中发挥了重要作用。整合政府公开电话资源，建立了首问责任制、首办负责制和服务承诺制。坚持重大项目安排和大额度资金使用集体决定，强化重大项目效能监察、重点事项提前介入监督。深入开展“做勤廉表率、促科学发展”主题教育活动，全面落实廉政建设责任制，惩治和预防腐败体系不断完善。

　　各位代表！一年来，面对跌宕起伏的外部环境，我们始终保持必胜的信念和勇气，经受了严峻考验，创造了新的业绩。成绩的取得，得益于党中央国务院、省委省政府和市委的坚强领导、科学决策；得益于各级各部门的密切配合、尽职尽责；得益于全市人民的凝心聚力、拼搏奉献；得益于历届市领导班子创造的环境、打下的基础。在此，我代表市人民政府，向全市人民和外地来青建设者，向离退休老同志，表示崇高的敬意！向人大代表、政协委员，各民主党派、各人民团体和各界人士，向中央和省驻青单位、驻青部队、武警官兵、公安干警，向积极帮助我市抗击浒苔灾害、筹办奥帆赛事的国家有关部委、省内外兄弟城市，向所有关心支持青岛建设发展的海内外朋友，表示衷心的感谢！

　　我们也清醒地看到，伴随宏观环境的重大变化，经济发展中一些深层次的体制性、结构性矛盾进一步凸显，政府工作还存在一些问题：一是产业结构调整任务艰巨，现代服务业发展滞后，自主创新能力不强，发展方式转变更加迫切；二是稳定就业形势严峻，农民持续增收难度加大，部分群众生活比较困难；三是城乡基础设施、环保设施、公共服务还不适应城市发展的需要，社会管理还有不少薄弱环节；四是安全生产形势不容乐观，基础工作还不牢固，重点领域的事故隐患还没有根本消除；五是一些政府部门为企业、为市民服务的主动性有待提高，部门之间的协调配合有待完善，一些政府工作人员的宗旨意识、工作作风有待加强，官僚主义和腐败现象仍然存在。对这些问题，我们将采取切实有效的措施，着力加以解决。

　　二、2009年政府工作的总体要求

　　各位代表！今年是新中国成立60周年，也是实施“十一五”规划的关键一年。政府工作的总体要求是：全面贯彻党的十七大和十七届三中全会精神，以邓小平理论和“三个代表”重要思想为指导，深入贯彻落实科学发展观，把保持经济平稳较快发展作为首要任务，积极作为、科学务实，全面实施“环湾保护、拥湾发展”战略，围绕“扩内需、促增长，调结构、上水平，重民生、保稳定”，着力加大投资、促进消费，加快发展方式转变和结构调整，全面深化改革、扩大开放，认真解决就业等涉及群众利益的实际问题，促进社会和谐稳定，实现经济社会又好又快发展。

　　经济社会发展的主要预期目标是：全市生产总值增长11%左右；地方财政一般预算收入增长8%左右；规模以上固定资产投资增长20%；社会消费品零售总额增长15%；城市居民人均可支配收入和农民人均纯收入增长8%左右；城镇登记失业率控制在4.3%左右；居民消费价格指数涨幅控制在4%左右；人口自然增长率控制在5％。以内；万元生产总值能耗下降5.04%，化学需氧量排放量下降5%，二氧化硫排放量不超过上年水平。

　　上述指标，除人口自然增长和节能减排约束性指标外，均是预期性和导向性的。特别是在世界经济增长明显减速、外部环境复杂多变、企业经营困难加重的形势下，实现上述目标有很大难度。我们将在实际执行中努力争取实现，力争完成得更好一些。

　　各位代表！今年，青岛的发展既面临着前所未有的挑战，也蕴含着重大的机遇。我们要看到，当前，全球金融危机在继续蔓延，国际经济环境在继续恶化，世界经济的不稳定、不确定因素和突发性风险明显增大。作为沿海开放城市，青岛面临的形势将更加严峻和复杂。我们必须时刻保持清醒的头脑，坚定信心、迎难而上，适时研究采取力度更大的政策措施，把扩大投资和消费作为保增长的主要途径，全力以赴促进经济平稳较快发展；把稳定就业和保障困难群体作为主要责任，千方百计改善城乡居民生活；把保持财税稳定增长作为主要任务，坚决压缩一般性支出，全力保法定增长、保社会民生、保重点建设。我们要看到，我国仍然处在重要战略机遇期，经济社会发展的基本面和长期趋势没有改变；经过改革开放30年的发展，抵御和应对危机的实力显著增强；奥帆赛、残奥帆赛的7年筹办和圆满成功，使青岛的影响力、竞争力明显提高，发展环境明显改善。我们必须抓住当前有利时机，解放思想、开拓进取，在城乡基础设施建设上加快推进，在发展方式转变和结构调整上有所作为，在企业改革重组和技术改造上明显见效，在利用国际国内两个市场、两种资源上走在前面，在提高政府效能、建设节约型机关上率先突破，为建设富强文明和谐的现代化国际城市奠定坚实基础，为建设经济文化强省作出积极贡献。

　　我们坚信，有中共青岛市委的坚强领导，有改革开放30年的雄厚基础，有国家扩大内需政策的强力带动，有全市人民的齐心协力，我们一定能够战胜各种困难和挑战，夺取经济社会发展的新胜利！

　　三、2009年的主要工作

　　根据上述总体要求和目标任务，着重做好十个方面的工作。

　　（一）着力扩大投资消费需求，增强对经济增长的拉动作用

　　积极用好、用足、用活国家扩大内需各项政策，加快推进和提前实施一批重大基础设施、民生与社会事业项目，不断扩大居民消费需求，努力保持经济持续增长。

　　切实抓好重点项目建设。今年，加快130个、总投资1170亿元在建项目进度，新开工81个、总投资1080亿元的重点项目，使项目总投资达到2250亿元。年内，力争开工建设城市地铁、黄岛至日照铁路、灰埠至蓝村铁路、铁路客运北站、董家口港40万吨矿石码头等基础设施项目，实施南车青岛扩能改造等产业项目，上汽通用五菱发动机、北船重工海西湾造船基地等50个项目竣工。加快小麦岛综合改造等旅游项目建设。做好新机场的前期规划论证。完善项目建设协调机制，构建“绿色通道”。严格审批程序和建设管理。坚持节约用地，优先保障重点项目用地需求，提高土地集约利用水平。

　　加大企业技术改造力度。落实国家重点产业调整振兴规划，制定实施家电电子、汽车机车等9大产业三年推进方案。出台加强企业技术改造的意见，引导企业用好增值税转型、进口设备免税等优惠政策，抓好青岛碱业节能、青啤扩能等项目，力争技改投资占工业投资的比重超过50%。

　　提高消费对经济增长的贡献率。努力增加城乡居民收入。出台鼓励消费的政策措施，稳定消费预期，培育消费热点，增强消费能力。加快建设5大商贸集聚区、10大商业网点，改建一批特色街，完善社区便民商业设施。通过扩大消费信贷、发展租赁业务、健全中介市场等措施，促进住房、汽车等大宗消费。全面启动农村消费市场，健全流通销售网络，引导大型商贸企业向农村延伸，抓好一批重点批发市场升级改造，实施“万村千乡市场工程”。继续推进“家电下乡”，落实财政补贴政策。整顿规范市场秩序，营造健康安全的消费环境。

　　繁荣发展现代服务业。把发展服务业作为扩大消费、促进就业的重要举措，按照重点发展“九大产业”、实施“五个结合”、培育“三个载体”的思路，加快形成以服务经济为主的产业结构。综合利用奥运资源，加大国内旅游促销力度，积极培育奥运旅游、工业旅游、乡村旅游、文化旅游等新热点，改善旅游环境和服务，创建中国最佳旅游目的地。大力发展会展经济，着力打造一批品牌展会，逐步实现会展业服务外包和市场化运作。制定金融业发展总体规划，加大金融机构引进力度，推动青岛银行跨区域设立分支机构，加快建设金融集聚区。优化物流业发展环境，加强与国内外知名物流企业的合作，积极争取增加国际国内航线，提高港口集疏运能力和空港货运中转量。加快建设胶州铁路集装箱中心站，高起点规划建设空港物流园区。促进科技信息、中介服务、总部经济等产业不断发展。服务业增加值占全市生产总值的比重达到45%以上。

　　（二）全面实施“环湾保护、拥湾发展”战略，提高城市集约发展的空间潜力

　　今年，要把实施“环湾保护、拥湾发展”战略放在政府工作更加突出的位置，把高新区基础设施建设作为加大投资、拉动增长的主攻方向，科学规划，集约建设，精细管理，早见成效。

　　坚持规划先导。突出规划的科学性、前瞻性和权威性，促进城市有序、均衡、协调发展。争取城市总体规划获得国务院批复实施。做好环湾区域功能布局、产业发展、土地利用、道路交通、生态环境和供电、供热、给排水等专业规划的修编实施。完善董家口港区规划。

　　坚持生态优先。落实生态市建设目标责任，全面提升环境质量。加大胶州湾海洋环境和湿地保护力度，对建设用地、海域和海岸线等空间资源进行战略储备。新建、完善沿海防护林20万亩。规划建设李沧滨海公园、浮山生态公园和太平山中央公园。实施崂山水库上游和李村河等市区10条河道截污整治。加快小涧西垃圾焚烧发电厂建设。努力创建国家生态园林城市和全国绿化模范城市。

　　坚持重点突破。开工建设高新区防风暴潮和防洪排涝工程，完成主要道路建设及景观绿化，实现核心区内主体干道基本联通、地下管网等基础设施综合配套。开工建设科技企业加速器和后勤保障中心，完成创业大厦主体工程，形成项目落户的基本条件。加快海底隧道、海湾大桥及接线、环胶州湾高速公路市区段拓宽改造等工程进度，做好新疆路高架、重庆路改造项目前期工作，完成滨海公路南段一期工程。强化质量意识、精品意识和保护意识，积极推进市南云南路片区、市北小港湾、四方滨海新区、李沧西部、胶州少海新城等重要节点的综合开发。完善老城区企业搬迁改造配套政策，启动10家企业搬迁改造。支持五市承接老城区企业转移，促进省级工业园区和6个工业功能区突破发展。

　　坚持设施配套。重点抓好“电、水、热、气”管网改造和完善。积极推进宁东送电胶州接收站、虎山变电站改造等工程。推进城区污水处理厂升级改造和配套管网建设，污水集中处理率达到85%。用两年时间，基本实现老城区主干道架空电线入地和污水集中处理。对市区50年以上的供水管网进行改造。加快华电热电联产、崂山东亿等热源项目建设，市区新增集中供热面积500万平方米，普及率达到60%。开工建设第二条入市输气主干管道和环胶州湾天然气管网。

　　坚持精细管理。强化城市管理的法制化、人性化和属地化，完善城市管理体制，推行数字化城市管理新模式。高标准开展城乡环境综合整治活动，有效治理建设工地扬尘及周边环境。推进道路交通智能化建设，优化城市交通组织管理。今明两年，大量地下管网设施将进行完善改造，要着力抓好工程进度、规范管理、交通疏导、舆论宣传等工作，争取广大市民的理解和支持。

　　（三）扎实推进农村改革发展，促进农民持续增收

　　全面贯彻党的十七届三中全会精神，落实强农惠农政策，保证市级财政对农业投入增长幅度高于经常性收入增长幅度，加快建设社会主义新农村，构建城乡一体化发展新格局。

　　巩固农业基础地位。进一步增加粮食直补、良种补贴、农机具购置补贴、农资综合补贴，落实粮食最低收购价格政策。推进国家大型商品粮基地和优质粮食产业化工程建设，抓好蔬菜、花生、果茶花卉三个百万亩示范区，建成一批标准化畜禽、水产养殖基地，保证粮食安全和主要农产品有效供应。健全现代农业产业技术体系，促进农业科技成果转化。积极推动农业产业化经营，培育扶持100个“一村一品”示范村、50户农业龙头企业和40家示范性农民专业合作社。

　　加强农村基础设施建设。对大沽河、墨水河及部分中小河道进行综合治理，对52座中小型水库进行除险加固，对大中型灌区实施续建配套与节水改造。加快农业综合开发，改造中低产田16万亩。推进国家中心渔港和一级渔港建设。完成1000个村“五化”工程。基本实现村村通油（水泥）路。大力发展农村沼气、秸秆气化等新能源。加大经济欠发达镇和集体经济薄弱村帮扶力度。

　　继续深化农村改革。稳定完善农村土地承包关系，做好集体土地所有权确权登记颁证工作，确保土地承包经营权证发放到户。坚持依法自愿有偿原则，引导农民多种形式流转土地承包经营权，发展适度规模经营。落实最严格的耕地保护制度和最严格的节约用地制度。做好集体林权制度改革和小城镇发展改革试点。

　　（四）统筹做好财政金融工作，加强经济运行调度

　　努力保持财政稳定增长。今年财政增支减收因素很多，保持收支平衡需要作出更大的努力。依法加强税收征管，完善税源控管体系，严格规范非税收入，严厉打击偷逃骗税行为。从紧安排财政预算，优化财政支出结构。对一般行政事业经费以2008年预算为基数压缩5%。加大审计力度，最大限度地提高财政资金使用效益。启动政府性债务管理制度试点。

　　充分发挥金融对经济增长的支持作用。加强产业政策与信贷政策的协调配合，引导金融机构扩大信贷投放，更好地服务实体经济。支持各类金融机构向县域延伸。推进农联社改制为农村商业银行，扩大村镇银行和小额贷款公司试点范围。鼓励民间资本、社会资本参与城乡基础设施和重大工程建设。充分发挥保险的融资和保障功能，积极发展住房、汽车和健康、养老等保险业务。支持大型企业集团设立财务公司。鼓励企业扩大直接融资比重，做好创业板上市准备工作。优化金融生态环境，有效防范和化解金融风险。

　　努力帮助企业渡过难关。强化扩大内需、经济运行等机构职能，发挥大企业服务直通车的作用，全面落实减免税费政策，积极帮助企业解决实际困难。继续推动工贸对接和直供配送，提高建设项目地产品配套率。做好能源、原材料供需衔接，确保煤电油运等稳定供应。加强对重点行业、企业和产品的监测分析，提高预测预警能力。落实支持民营经济、中小企业发展的政策措施，发挥专项资金政策导向作用，加强信用担保等社会化服务体系建设，扩大政府采购供应商范围，鼓励中小企业向“专、新、特、精”方向发展。

　　（五）深入推进改革开放，增强经济社会发展活力

　　加快重点领域和关键环节改革。积极推进企业并购、重组和产业整合，提高产业集中度和资源配置效率。深化国有资产经营公司改革。贯彻实施《企业国有资产法》，加强国有资本经营预算管理。创新监管体制机制和监管方式，规范完善企业法人治理结构。深化投融资体制改革，优化政府经营类投资公司资产结构，强化投融资功能和资金使用安全。推进增值税转型改革，落实统一内外资企业城建税等政策。做好成品油价税费改革配套工作，逐步理顺资源性产品价格。抓好经济技术开发区省级综合配套改革试点。按照国家和省的部署，做好行政管理体制改革。

　　坚定不移推进对外开放。全面落实促进外经贸发展的各项政策。实施市场多元化战略，巩固稳定传统市场，大力开拓新兴市场，扩大先进技术设备、关键零部件和原材料进口。完善贸易摩擦应对机制，积极支持企业参与国内外重点展销活动。确保前湾保税港区一期封关运营，形成开放新优势。跟踪把握当前国际产业转移和投资新趋势，着力引进先进技术和先进制造业、现代服务业项目。抓住与台湾海空直航的有利时机，加强对台经贸合作。支持有条件的企业开展对外投资、承包工程和外派劳务，低成本收购国际品牌、营销网络和研发中心。扩大国内经济交流，加强与省内城市的合作。坚持按制度运行、按程序操作、按章程办事、按规范服务，进一步优化开放环境。做好外事、侨务工作，积极拓展对外交流合作的渠道和领域。

　　（六）加强自主创新和节能减排，促进发展方式转变

　　全面提高自主创新能力。围绕建设创新型城市，加大对全社会各类创新活动的支持力度。推进国家深海基地、海洋科学与技术国家实验室、企业国家实验室、工程技术研究中心建设，建立市级科技研发服务中心和资源共享平台。以信息化推动工业化，带动先进制造业、现代服务业融合发展。实施家电电子等领域18项关键技术攻关,加快建设高速列车等10大产业化基地，抓好1000个技术创新项目。积极推动医疗卫生、环境保护、交通安全等社会民生领域的技术研发。优化科技专项经费使用，提高政府性资金的投入效益。实施品牌战略和标准化战略，主要工业品采标率达到80%。加强知识产权保护，争创国家版权保护示范城市。

　　实施人才强市战略。制定高层次优秀人才来青创新创业发展实施细则，加大人才引进力度。积极探索创新型人才培养模式，实施优秀创新团队重点扶持计划。完善高技能人才、首席技师评选办法和优秀人才选拔推荐奖励制度，健全医疗保障、子女就学等配套政策，努力营造人尽其才、人才辈出的良好环境。

　　继续强化节能减排和环境保护。严格落实建设项目能耗审核制度。加强对110家用能单位目标责任考核和100家排污企业环境监管。推进10大节能工程和既有建筑、公共机构节能改造，淘汰城市道路和公共场所低效照明产品。对35户重点企业实施强制性清洁生产审核，加大小火电关停、燃煤锅炉改造力度，建立淘汰落后产能企业名单公告制度。加快20个生态环保园区建设，推动100家单位循环经济试点工作。

　　（七）突出抓好就业和社会保障，不断改善城乡群众生活

　　着眼于提供均等化的公共服务、普惠性的社会保障、公平性的发展机会，认真解决涉及群众利益的难点热点问题。确保完成今年为人民群众办好的9件实事。

　　努力稳定和扩大就业。实施更加积极的就业政策，坚持以项目扩大就业、以创业带动就业、以培训促进就业，全力保持就业稳定。健全失业预警应急机制，对经营困难的企业，给予岗位补贴和社会保险补贴，实施在职培训援助计划，引导企业稳定劳动用工，履行社会责任。实施高校毕业生就业服务行动计划，完善离校未就业毕业生见习制度，鼓励到基层、农村和中小企业就业。设立创业扶持资金，建立创业孵化基地，支持高校毕业生和社会各类人员自主创业。做好公益性岗位开发和储备，帮助就业困难群体特别是城镇“零就业家庭”有效就业。有针对性地开展农民工用工余缺对接和技能培训。加强劳动保障监察，维护劳动者合法权益。

　　加大城乡群众生活保障力度。完善最低生活保障制度，提高优抚对象抚恤补助标准，落实家庭困难学生资助政策，加强对低收入家庭的补贴和救助。继续提高企业退休人员基本养老金水平，平均每人每月由1234元提高到1386元。切实做好抗日战争、解放战争参加革命老同志的保障。提高失业人员保险金标准。扩大新型农村合作医疗报销范围，降低农民医疗费用自费比例。扩大新型农村社会养老保险覆盖面。研究探索城乡养老、医疗保险的转移接续办法。

　　改善城乡群众居住出行条件。坚持“政府有所作为、重点保障低收入家庭、土地出让收益回馈民生”的原则，加大保障性住房建设力度,市区建筹廉租住房3000套，经济适用住房5000套。启动“两改”项目12个，改造筒子楼40栋，整治旧住宅区4片，受益居民1.4万户。提高低保家庭廉租住房租金补贴标准。年底前，力争建成保障性住房1.29万套，使市区低收入住房困难家庭实物配租、配售达到64%。竣工“两改”项目25个，回迁居民2.4万户。今年起，以区市政府为主导，开展以农民宅基地置换经济适用住房、向无宅基地农民配售经济适用住房的试点。适应城市布局变化，适时开辟调整公交线路，新建2处市区公交停车场和8个农村客运站，逐步推行近郊线路公交化，切实提高公交运行质量和服务水平。

　　各位代表!越是困难的时候，越要关注民生。尽管当前经济发展面临诸多挑战，但是无论困难多大、财力多紧、任务多重，我们坚持富民优先的理念不会变，财力惠民投入的强度不会降，推进民生工程的力度不会减，尽最大的努力改善民生，让城乡群众安居乐业有保障、分享更多的发展成果！

　　（八）推动社会事业全面发展，努力提升城市文明

　　认真办好人民满意的教育。制定我市中长期教育改革和发展规划纲要。全面加强素质教育，促进义务教育优质均衡发展。免除农村义务教育阶段公办学校寄宿生住宿费。加大中小学校舍抗震加固和危旧房屋改造力度，抓好安全应急演练。加快浮山新区等新建居民区配套学校建设。整合职业教育资源，推进职业学校布局、专业调整和校内实训基地建设。促进高等教育内涵发展，强化重点学科建设，充分发挥高校在人才培养、科技成果转化、服务经济社会中的作用。重视学前教育、特殊教育，加强成人教育和民办教育。

　　积极推进健康城市建设。深化医药卫生体制改革。开工建设北部医疗中心，完成国医堂、妇女儿童医疗保健中心主体工程。落实国家基本公共卫生服务项目，完善社区卫生和农村卫生配套政策，健全基本医疗卫生制度。全面加强重大疾病防治和卫生应急管理，提高医疗质量和服务水平，构建和谐的医患关系。稳定现行生育政策和低生育水平，提高出生人口素质。

　　努力推动文化繁荣发展。创新文化体制机制，大力发展新闻出版、广播电视和文化创意产业，加快建设影视传媒基地、国家动漫创意产业基地、市图书大厦，基本建成青岛大剧院，确保奥帆博物馆竣工开放。扶持一批文化企业做大做强，培育一批体现青岛特色的文艺精品，办好青岛音乐节、全国小提琴演奏比赛和全民读书月等文化活动。加强文化遗产保护。做好网络文化建设与管理。促进科学普及，繁荣发展哲学社会科学。

　　不断加强精神文明建设。以“塑造美好心灵，建设美好家园”为主题，推进新一轮文明城市创建工作。实施“四心”工程，精心打造“文明赢得尊重”等精神文明建设活动品牌，引导市民从自我做起，着力提高思想道德和文明素质。实施城区“文明畅通示范工程”和农村精神文明建设“四个一”工程。积极做好社会志愿服务工作。加强社会诚信体系建设。探索建立城市文明指数日常测评和结果定期公布机制，推进文明创建常态化、科学化、制度化。

　　全面发展其他各项事业。高水平办好第十一届全运会青岛赛区各项赛事。建成市体育馆、游泳跳水馆。组织好沃尔沃、克利伯等大型帆船赛事青岛站活动，推动帆船运动深入开展，打造“帆船之都”城市品牌。广泛开展“全民健身与全运同行”活动。办好第二届市运会。加快发展老龄事业和老龄产业，完善社会化养老服务体系，开工建设老年活动中心。积极发展妇女儿童、青少年和社会福利、公益慈善、残疾人、红十字事业。依法推进民族、宗教事务管理，做好档案、史志、仲裁等工作。加强国防教育，做好人民防空、民兵预备役和复员转业军人安置工作，支持驻青部队和武警部队建设，广泛开展“双拥”活动，巩固军政军民团结。以对四川灾区人民高度负责的精神，加快对口援建乡镇群众基本生活设施和公共服务设施的恢复重建，确保完成全年援建任务。

　　（九）深化平安和谐创建工作，营造安全稳定的社会环境

　　各位代表！今年安全生产工作压力很大，社会稳定任务艰巨。我们必须把人民群众的生命安全放在至高无上的位置，加强重点行业、领域的安全生产，加强食品药品安全，加强公共安全应急体系建设和社会治安综合治理，努力保持社会安定祥和。

　　全力抓好安全生产。开展“安全生产责任落实年”活动。强化政府监管责任，全面推行网格化管理，健全乡镇、街道办事处专职安监队伍，严格行政问责。强化企业主体责任，切实提高安全自律意识和安全防范能力。建立安全检验检测认证与安全生产责任挂钩制度。切实抓好以交通安全、火灾防范、建筑工地为重点的安全专项整治，严格执行安全设施“三同时”规定，坚决遏制重特大事故发生。

　　强化食品药品质量安全。围绕创建“全国食品最安全城市”，进一步健全食品药品安全保障体系，深入开展食品药品安全专项整治。加快检验检测支撑平台建设，强化源头监控和市场监管，实行严格的质量追溯和召回制度、市场准入和退出制度。

　　切实维护社会稳定。积极推进城乡和谐社区建设，进一步改进社会管理和服务方式，最大限度地整合各种社会资源和力量，共同参与社会管理。完善“大接访”工作机制，坚持领导干部特别是主要领导处理和接待群众来信来访制度，着力解决突出的信访问题，引导群众理性合法表达利益诉求。以群众安全感和满意率为标准，健全社会治安防控体系，完善经常性的治安问题排查整治机制。加强社区警务和“五五”普法工作，改进流动人口管理服务。以“打黑除恶”为重点，严厉打击各类严重刑事犯罪活动。高度重视城市公共安全，增强全社会风险防范意识，加强社会安全应急体系建设，有效预防和处置各类突发事件，做好对浒苔、赤潮等自然灾害的预警防范，提高城市的公共安全保障能力。

　　（十）加强政府自身建设，营造良好发展环境

　　特殊时期、特殊考验，需要政府有更大的作为，需要政府工作人员付出更大的努力。我们将以开展深入学习实践科学发展观活动为契机，强化政府服务，推进管理创新，改善发展环境，加快建设服务型政府。

　　认真开展深入学习实践科学发展观活动。按照省、市委的统一部署，围绕“提高思想认识，解决突出问题，创新体制机制，促进科学发展”的目标，认真抓好学习调研、分析检查、整改落实、总结测评等各项工作。进一步加强理论学习和调查研究，切实增强政府工作人员贯彻落实科学发展观的自觉性和坚定性，着力解决影响制约科学发展的体制机制，着力解决经济社会发展中的突出矛盾，不断提高推动科学发展、促进社会和谐的能力，努力达到“党员干部受教育、科学发展上水平、人民群众得实惠”的要求。

　　坚持依法行政。自觉接受人大及其常委会的法律监督和工作监督，主动接受政协的民主监督，认真听取民主党派、工商联、无党派人士和各人民团体的意见。接受社会公众监督和舆论监督。办好人大代表议案、建议和政协提案。加强行政立法，坚持重大行政决策合法性审查制度。严格行政执法责任制和许可告知制，完善行政复议制度和过错追究制度。

　　着力提高行政效能。完善政府服务管理、社会组织动员的体制机制，加强部门协调配合。推进行政审批制度改革，实行“集中审批、授权到位”模式。充分发挥行政审批服务大厅作用，扩大网上全程办理及市区两级联动办理，建立审批事项清理和流程优化动态机制，依法从严控制新增收费，使青岛成为政府效能最高、行政成本最低的城市之一。大力推行电子政务，加快政府工作流程再造。依法推进政府信息公开，提高政务公开电话、网上政府信箱便民程度，增强政府工作的透明度和公信力。坚持重大事项集体讨论决定制度，完善社会公示、听证和专家咨询、评估机制，使政府做出的部署、出台的政策更加符合群众意愿。健全行政问责制度，强化责任追究，建立科学合理的目标绩效考核体系，推进政府绩效管理。

　　切实加强廉政建设。按照胡锦涛总书记在中纪委十七届三次全会上提出的“六个着力”要求，牢固树立正确的事业观、工作观、政绩观。认真执行领导干部廉洁自律规定。以完善惩治和预防腐败体系为重点，努力从源头上形成拒腐防变教育长效机制、反腐倡廉制度体系、权力运行监控机制。加强对政府部门执行财经法规、主要领导经济责任的审计监督。健全政府重大投资项目公示、重大项目效能监察、重点事项提前介入等制度。建设综合电子监察平台和公共资源交易中心。各级政府要厉行节约，勤俭办事，深入开展创建节约型机关活动。加大商业贿赂治理力度，坚决纠正损害群众利益的不正之风，努力营造风清气正的政务环境。

　　各位代表！走过改革开放30年光辉历程，我们又站在新的历史起点。困难和挑战考验着我们，责任和使命激励着我们。让我们更加紧密地团结在以胡锦涛同志为总书记的党中央周围，高举中国特色社会主义伟大旗帜，深入贯彻落实科学发展观，在中共青岛市委的领导下，坚定信心，振奋精神，开拓进取，努力做好全年的各项工作，以优异成绩迎接新中国成立60周年！
